# Epigenetic changes: a common theme in acute myelogenous leukemogenesis

**DOI:** 10.1186/1756-8722-6-57

**Published:** 2013-08-13

**Authors:** Soraya E Gutierrez, Francisco A Romero-Oliva

**Affiliations:** 1 Departamento de Bioquimica y Biologia Molecular, Facultad de Ciencias Biologicas, Universidad de Concepcion, Casilla 160 C, 4089100, Concepcion, Chile; 2 Centro de Estudios Cientificos SINDAB, Santiago, Chile

## Abstract

Acute myeloid leukemia (AML) is a rather common disease, characterized by the presence of a clonal population of hematopoietic progenitor cells with impaired differentiation. Although traditionally AML has been considered the result of genetic alterations, more recently experimental evidence have demonstrated that epigenetic modifications are important in development and maintenance of leukemia cells. In this review we summarize current scientific knowledge of epigenetic alterations involved in leukemogenesis. We also highlight the developing of new technological strategies that are based on epigenetic processes and have been registered as Patents of Inventions in the United Nations dependent World Intellectual Property Office (WIPO) and the main Patent offices worldwide.

## Introduction

The World Health Organization (WHO) defines acute myeloid leukemia (AML) as a heterogeneous clonal disorder of hematopoietic progenitor cells (“blasts”), which exhibit impaired maturation [1]. AML is the most common acute leukemia in adults and, in the absence of treatment, this alteration in blood cells leads to dead typically within 1 year of diagnosis mainly by infection, bleeding, or organ infiltration. Until now, treatment of AML consists of cytotoxic “chemotherapy” and might cure 20–75% of patients younger than 60 years depending primarily on leukemia-cell cytogenetics. However, in elderly patients despite achievement of reasonable complete response rates (CR rates 35%-55%), intensive chemotherapy is associated with a high incidence of 4-week mortality and with 3- to 5-year survival rates of < 10% [2-4]. Currently is estimated that 7.820 men and 6.770 women will be diagnosed and 10.370 (men and women) will die of AML in 2013 in US. The majority of the AML cases are associated with nonrandom chromosomal translocations. Although over 700 recurrent aberrations have been described associated with the AML phenotype, the four more common are: t(15; 17)/PML-RAR, t (8;21)/AML1-ETO. Inv(16)/core binding factor (CBF)b-MYH11, 11q23 and mixed lineage leukemia (MLL)-fusion proteins.

Traditionally AML has been considered the result of genetic alterations leading to irreversible defects of critical gene functions such as proliferation, differentiation, apoptosis and gene transcription associated to leukemogenesis. The mutated genes are often grouped in two classes: genes which confer a growth advantage by activating downstream effectors of various signaling pathways (including members of the signal transducer and activator of transcription (STAT), PI3K and RAS–MAPK pathways) and genes which alter the expression of key transcriptional targets in myelopoiesis (e.g. PML, RUNX1, MLL). In all cases, the end result is that the affected cell loses the ability to differentiate and to respond to cell proliferation regulators. DNA in human cells is found associated to proteins forming the chromatin. Packing eukaryotic genomes into high-order chromatin structures is critical for controlling most, if not all, processes derived from DNA. The minimal repeating unit of chromatin is the nucleosome, comprised of 147 base pairs wrapped around a histone octamer core [5]. In comparison to “naked” DNA, nucleosomal DNA is less accessible for DNA-binding proteins such as transcription factors, DNA replication and DNA repair complexes. Nucleosome cores are connected by linker DNA sequences of variable length to give an average DNA length of approximately 200 bp. Arrays of 11-nm nucleosomes are thought to condense into approximately 30-nm fibers. A fifth histone, the linker histone H1, is structurally distinct from other histones and it has been shown that facilitates compaction of nucleosomes into 30-nm fibers and higher order chromatin structures. Therefore, nucleosomes serve as a potent mechanism for controlling transcription and other processes that utilize DNA as template [6]. The changes between tightly packed DNA (heterochromatin) and exposed DNA (euchromatin) are coordinated through modifications of the nucleosome structure either by DNA methylation, histone post-translational modifications (e.g. acetylation, methylation) or by ATP-dependent chromatin remodeling complexes (a group of protein complexes that can slide nucleosomes on DNA or bring about the exchange or eviction of the histones). These heritable changes in DNA packing that regulate DNA transcriptional activity are collectively known as epigenetic modifications. In the past few years, several reports have linked the development of the AML phenotype to epigenetic alterations [7-9]. For instance, recent genome-wide and candidate-gene studies have identified somatic alterations in genes that encode proteins regulating DNA methylation and post-translational histone modifications. These data suggest that somatic alterations in epigenetic regulators are a common genetic event in AML and contribute to hematopoietic cell transformation. In fact, epigenetic changes play a crucial role in the regulation of gene expression and several studies have reported epigenetic abnormalities occurring within signaling pathways regulating proliferation, migration, growth, differentiation, transcription, and death signals that may be critical in the establishment and progression of malignancies [10]. This data are underscored by recent studies which suggest that mutations in a subset of epigenetic regulators, including TET2 (Ten eleven Translocation protein 2), ASXL1 (Additional Sex Combs protein 1) and DNA methyl transferase 3a (DNMT3a) [11-14], are associated with poor overall survival of AML patients; therefore, defining a new subset of high-risk leukemia that is in need of novel, mechanism-based therapies. More importantly, because the epigenetic modifications are reversible, therapies based in epigenetic modifiers hold the promise of being highly effective. In this review we will discuss recent data implicating epigenetic alterations in the pathogenesis of AML, risk stratification and therapeutic of patients with myeloid leukemia; patents applications presented worldwide related with epigenetic modifiers or modifications are also summarized.

### Epigenetics

As mentioned above, epigenetics is defined as the study of heritable changes in gene expression that are not due to modifications in the DNA sequence. Epigenetic changes can be established through multiple molecular mechanisms that include DNA methylation, histone modifications and more recently, the action of small RNA that do not codify for a protein or polypeptide but can regulate gene expression, known as non-coding RNAs (ncRNA). All of these different modifications are closely interrelated and can influence each other. Interestingly, epigenomic profiling studies of patients with AML have revealed alterations in DNA methylation [15,16], oxidized derivatives of methylated cytosines [17], and alterations in histone post-translational modifications such as lysine methylation [18,19], phosphorylation [20], and acetylation [21,22], suggesting a fundamental role for these modifitions in AML pathogenesis.

### DNA methylation

In eukaryotes, ranging from plants to humans, DNA methylation is found exclusively at cytosine residues, which most commonly are forming part of a CpG dinucleotide. The CpG dinucleotides are not homogeneously distributed throughout the genome, normally they are clustered in short DNA regions highly rich in CpG that are known as CpG islands, and in highly repetitive regions such as centromers and retrotranposons [23,24]. Approximately 60% of the human genes have CpG islands in their regulatory regions (promoters) [25]. In the human genome there are about 13,000 CpG islands that are constitutively unmethylated [26]. However, a small but significant proportion of all CpG islands become methylated during development and when this happens the associated promoter is stably silent. Developmentally programmed CpG-island methylation of this kind is involved in genomic imprinting and X chromosome inactivation [23]. In contrast, most of the genomic CpG dinucleotides are methylated in all tissues and is thought to play a role in suppressing recognition of spurious intragenic DNA-binding sites to protect the cell from uncontrolled transcriptional activity and genomic instability [27].

DNA methylation results in stabilization of transcriptional repression and loss of gene function when present in promoters [23,28,29]; however, is associated with active expression when present into the genes (exons + introns) [30,31]. It has been suggested that these divergent relationships of DNA methylation with gene expression could be driven by a common role of DNA methylation in the stabilization of nucleosomes and in the occlusion of the transcriptional initiation site. Likewise, the introduction of a methyl group to the DNA can directly alter the binding of transcription factors [23,32,33], further buffering against recognition of intragenic DNA-binding sites in actively expressed genes. Alternatively, DNA methylation may create new binding sites for proteins with methylation recognition domains which in turn induce gene repression by recruiting histone deacetylases (HDACs) [34,35].

DNA methylation occurs within CpG dinucleotides through addition of a methyl group at the 5′ position of the cytosine ring, forming 5-methyl cytosine, in a reaction catalyzed by enzymes known as DNA methyl transferases (DNMTs) [36]. There are three principal DNA methyltransferases: DNMT1, DNMT3a and DNMT3b. DNMT1 is the primary maintenance enzyme that preserves existing methylation patterns following DNA replication by adding methyl groups to corresponding daughter strands at the hemi-methylated CpG sites. DNMT3a and DNMT3b are methyltransferases that preferentially target unmethylated CpGs to initiate *de novo* methylation and therefore, they are highly expressed during embryogenesis but minimally expressed in adult tissues. A fourth family member, DNMT-3 L, lacks intrinsic methyltransferase activity; however it facilitates methylation of retrotransposons by interaction with DNMT3a and 3b [37].

DNA methylation is the most studied epigenetic alteration in cancer and was the first epigenetic alteration to be connected to its development [38,39]. CpG island methylation is now widely recognized to be associated with cancer-related changes in gene expression. These changes are produced mainly through three different mechanisms: hypomethylation, loss of imprinting, and hypermethylation. As mentioned above, in addition to regulation by DNA methylation itself, methylated DNA binding proteins (MBDs) can bind to methylated cytosine, and sequentially form a complex with histone deacetylase (HDAC) leading to chromatin compaction and gene silencing [40].

Global hypomethylation can lead to chromosomal instability, mutations and reactivation of various oncogenes. For instance, DNMT1 is responsible for the establishment of the DNA methylation pattern during DNA synthesis, and its deficiency in cells may lead to global hypomethylation. Another common alteration observed in cancer cells is DNA hypermethylation of promoter-associated CpG islands of tumor suppressor genes, which could serves as a surrogate for point mutations or deletions to cause transcriptional silencing of these genes [41,42].

A detailed study on the genomic methylation landscape of AML [43] have identified 16 distinct methylation patterns; each of these DNA-methylation AML subtypes displayed a unique epigenetic signature when compared with normal bone marrow CD34+ cells. These patterns most accurately overlapped with the currently known molecular subtypes of AML while simultaneously revealed the existence of additional epigenetic differences among patients. In fact, three of the 16 patients clusters correspond to AML subtypes defined by WHO: t(8;21), inv(16) and t(15;17). Although this finding was perhaps expected, it is an important validation of the use of large-scale genome-wide DNA methylation profiling technology. Moreover, their data are consistent with the hypothesis that each of these fusion oncoproteins can drive the establishment of a specific epigenetic patterning in hematopoietic cells. Notably, this analysis also defined five new AML subtypes that could not be explained by any known morphologic, cytogenetic, or molecular feature. In fact, each of these AML subtypes displays an unique and significantly different epigenetic signature when compared to normal CD34+ control cells and a significant difference in patient survival was observed between these novel AML subtypes. Taken together, these results indicate that DNA methylation profiling identified clinically relevant AML subtypes that cannot be captured by any of the currently available diagnostic method.

Surprisingly, in this work the authors show that not all the identified methylated genes are repress at the expression level suggesting that other factor(s) may be still needed to silence them or alternatively that promoter methylation-mediated silencing is overcome by another mechanism [43].

The methylation profiles identified by Figueroa et al [43] support the idea that AML patients can be identified by a core set of genes that are commonly methylated in AML cells compared with normal hematopoietic cells. Moreover and more importantly, their results suggest that genomic methylation markers can be used for improved molecular classification and may have prognostic value for AML.

A novel class of point mutation described in AML affects the isocitrate dehydrogenase (IDH) genes [44-46]. The two enzymes affected (IDH1 and IDH2), normally catalyze the oxidative decarboxylation of isocitrate to α-ketoglutarate and reduce NADP to NADPH. The point mutations cause loss of native enzymatic activities and confer novel enzymatic activity, catalyzing the conversion of α-ketoglutarate to 2-hydroxyglutarate (2-HG), an oncometabolite. 2‐HG is present at markedly elevated levels in the serum of patients with AML harboring IDH1 or IDH2 mutations, suggesting that 2‐HG could be a biomarker for IDH-mutant AML [46]. All known mutations involve arginine (R), in codon 132 of IDH1 or codon 140 or 172 of IDH2. Although mutations of R140 in IDH2 exclusively result in the substitution of arginine to glutamine (IDH2‐R140Q), mutations affecting IDH1‐R132 or IDH2‐R172 consist of a broader range of amino-acid substitutions (IDH1‐R132H, IDH1‐R132C, IDH2‐R172K, IDH2‐R172M and IDH2‐R172S). IDH1 (R132) and IDH2 (R140) mutations are frequently accompanied by normal cytogenetics and nucleophosmin (NPM1) mutation, whereas IDH2(R172) is frequently the only mutation detected in AML. Moreover, mutational and methylation studies of a large cohort of AML patients demonstrate that IDH and TET2 mutations are mutually exclusive in AML and that patients with mutation in either IDH or TET2 genes exhibit similar methylation profiles characterized by global promoter hypermethylation [47]. Subsequent biochemical studies demonstrated that 2-HG inhibits TET family members, as α ketoglutarate is an essential cofactor of TET, and therefore its activity is reduced by the production of 2-HG [48,49]. TET proteins are dioxygenases that catalyze the conversion of 5 methyl cytosine (5mC) to 5 hydroxyl methyl cytosine (5hmC) [50]. Tet proteins can further oxidize 5hmC to generate 5-formylcytosine (5fC) and 5-carboxylcytosine (5caC), which can then be removed from the genome by thymine-DNA glycosylase (TDG) [51-53]. This suggests that 5hmC may act as a DNA demethylation intermediate. TET2 is often silenced or mutated in myelo proliferative disease (MPD), myelodysplastic syndrome (MDS), chronic myelomonocytic leukemia (CMML) and lymphomas suggesting a tumor suppressor role for TET2 in myeloid tumors, MPD and MDS. 5hmC not only impairs the binding of 5mC binding proteins [54], but has its own binding proteins [55] and shows unique distribution patterns in the genome [56,57], suggesting that 5hmC may serve as an epigenetic mark with different regulatory functions.

Mutations in TET2, IDH1 or IDH2 seem to have clinical relevance to risk stratification and/or therapeutic relevance in patients with AML. In fact, mutations in TET2 confer adverse overall survival in intermediate risk-subset of AML regardless of fms-related tyrosine kinase gene (FLT3) mutational status [58]. However, prognostic impact of IDH1/2 mutations seems to vary according to the specific mutation and also depends on the context of concurrent mutations of other genes. For instance, IDH2(R172) mutations confer a poor prognosis in AML patients, while IDH1(R132) mutation may predict poor outcome only in a subset of patients with molecular low-risk AML [59]. As mentioned above, the mutations in IDH generate a new enzymatic activity which result in 2-HG production affecting the enzymatic activity of the TET family; however, there are other α ketoglutarate-dependent enzymes that may also be inhibited by 2-HG affecting the cell epigenetic state including members of the jumonji-domain-containing (JMJC) family of histone lysine demethylases, which demethylate lysines 9 and 36 of histone H3 (H3K9 and H3K36) [60].

More recently, two reports found that the (R)-enantiomer of 2-HG ((R)-2HG) may actually serve as a cofactor for the activation of the Egl-Nine (EGLN) family of prolyl hydroxylases (EGLN1–3) [61,62]. In both reports, the authors found that oncogenicity of IDH1 mutants in neural and hematopoietic cells may depend on the increased activity of the EGLN family, which normally mark the oxygen-labile subunit of the transcription factor hypoxia-inducible factor (HIF) for proteasomal degradation. These data suggest that therapeutic targeting of the EGLN family may also be useful in the treatment of IDH-mutant AML.

### Histone modifications

**Figure 1:**
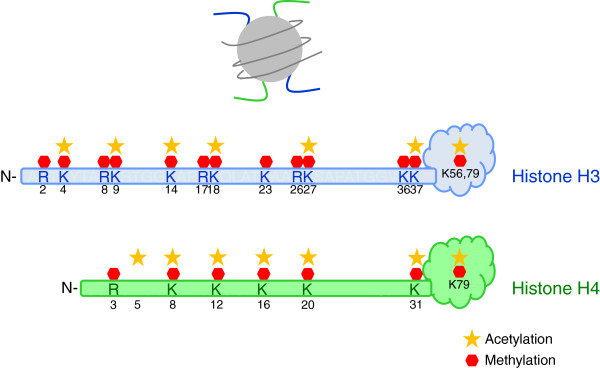
**Histone post-translational modifications.** Top panel correspond to a diagrammatic representation of a nucleosome showing histone H3 and H4 N-terminus (histone tails), which extend from the nucleosome particle. Bottom panel show some of the specific amino-acid residues that are methylated and/or acetylated in the N-terminus of histone H3 and H4 as well as some of the residues modified in the histones globular domain.

**Figure 2:**
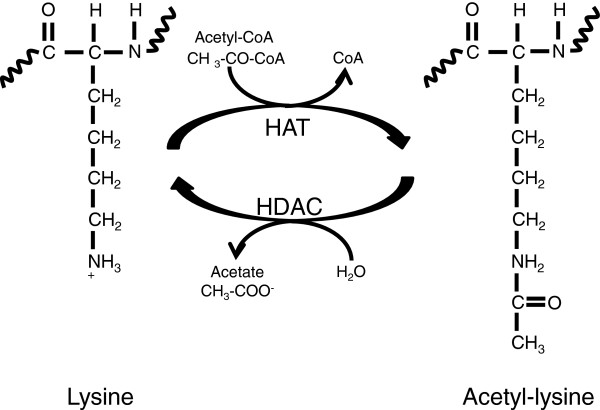
**Histone acetylation.** Histone acetyltransferase complexes (HATs) catalyze the transfer of an acetyl group from acetylCoA to the ϵ-amino of a lysine residue. Removal of an acetyl group from a ϵ-N-acetyl lysine amino acid on a histone protein is carried out by the histone deacetylase complexes (HDACs). Curly lines represent the rest of the protein molecule.

**Figure 3:**
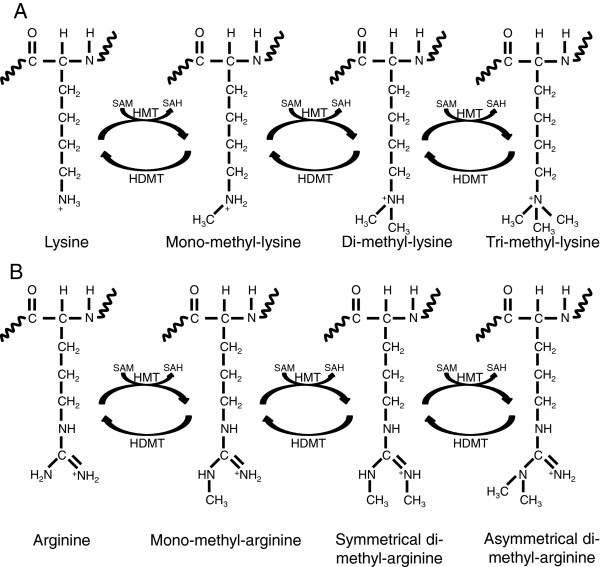
**Histones can only be methylated on lysine (K) and arginine (R) residues. A)** Lysine is able to be mono-, di-, or trimethylated with a methyl group replacing each hydrogen of its NH3+ group. **B)** Arginine, with a free NH2 and NH2+ groups, is able to be mono- or dimethylated. Arginine methylation can be asymmetric on the NH2 group or symmetric with one methyl on each group. Specific histone methyl transferases (HMT) and histone demethylases (HDM) catalyze these processes. Curly lines represent the rest of the protein molecule.

The major sites for histone posttranslational modifications are located in the N-termini of the histone proteins, known as ‘histone tails,’ which extend from the histone octamer of the nucleosome core (Figure 1). Histone may undergo several post-translational modifications, which can alter chromatin dynamics either by providing recognition sites for specific proteins (or protein complexes) or changing the nucleosome structure by altering electrostatic charge [63]. Histone modifications include acetylation, methylation, phosphorylation, sumoylation, ubiquitination, ribosylation and also proline isomerization. In most cases, the enzymes that carry out these functions are part of multiprotein complexes involved in some aspect of gene regulation or other genomic functions. According to the experimental evidence available, most of the histone tails modifications appear to have little effect in the internal structure of the nucleosome core [64]; however, they do affect interactions of nucleosomes with transcription factors and other nucleosomes. More recently, the characterization of bulkier histone modifications or modifications within internal histone domains has been suggested to potentially impact nucleosome structure [65]. Among the histone covalent modifications, the most studied are the acetylation in lysine (Figure 2) and the methylation in lysine and arginine residues (Figure 3) of histones H3 and H4, mainly in relation to their role in transcriptional regulation. In general, while histone acetylation is related to open chromatin structure, and therefore activation of gene transcription, histone methylation is found mostly associated with closed chromatin and transcriptional repression.

The enzymes that participate in establishing these histone modifications are the histone acetyltransferases (HAT) and the histone methyl tranferases (HMT) complexes, respectively; while the removal of the modifications are carried out by histone deacetylases (HDACs) and histone demethylases (HDMTs). Although the heritability of histone modifications themselves is not completely established, a great number of epigenetic cell memory proteins that have been implicated in human disease have also turned out to be enzymes that are involved in modify or recognize histone modifications [21,66-68].

### Histone acetylation

In addition to their effect on histone-DNA interaction, individual histone modifications can enhance or decrease the presence of another modification on a target gene, they can also interact with signaling pathways and with other epigenetic regulators such as DNA methylation, and noncoding RNAs (ncRNAs). This experimental evidence has led to the proposal of a histone code, which would be read by specific proteins or proteins complexes for the epigenetic regulation of gene expression [69,70]. Central to the establishment of the histone code, is the activity of HAT and HDAC enzymes. In fact, some HATs are recurrent components of fusion oncoproteins generated by chromosomal rearrangement in leukemia, for example t(8;16)(p11,p13) associated with AML generate the fusion protein MOZ-CBP which is composed for two HAT enzymes: MOZ (MOnocytic leukemia Zinc-finger protein) and CBP (CREB-Binding Protein). The different fusion proteins contribute to leukemic transformation most likely by a mechanism involving mistargeted histone acetylation and thus aberrant activation of gene expression [71,72]. Moreover, in a recent study, inactivating mutations within the HAT domain of CBP have been found in approximately 18% of relapsed AML cases, suggesting that the impaired HAT activity could be linked to resistant to therapy in AML [22].

It has also been proposed that aberrant recruitment of HDACs to target genes owing to oligomerization of a chimeric transcription factor could be a mechanism underlying a broader group of AMLs. Indeed, the AML1-ETO fusion protein was demonstrated to act through an aberrant HDAC-recruiting mechanism that leads to the block of hematopoietic differentiation [73]. Altered distribution of HDAC1 in AML and consequent specific pattern of chromatin modifications at hematopoietic genes could also be associated with the patient’s outcome, and thus be a tool to improve prognosis prediction [74]. Interestingly, the implications of HDACs in leukemia is not limited to their aberrant recruitment by fusion proteins [75-77], as alterations in the expression of various HDAC isoforms have also been associated with the patient’s prognosis [78].

### Histone methylation

MLL (Mixed Lineage Leukemia protein) is a histone methyltransferase required for the epigenetic maintenance of gene activation during development [79-81] and is also mutated in a subset of aggressive acute leukemias (both ALL and AML) [82]. MLL maintains gene activation in part by methylating histone 3 on lysine 4 [83,84]. The most common leukemogenic MLL mutations are chromosome translocations that fuse the N-terminus of the MLL gene in-frame with any of more than 70 different partner genes producing novel MLL fusion proteins [85,86]. Interestingly, the leukemias caused by MLL fusion proteins have very few additional genetic mutations [87-90], suggesting that the formation of the fusion protein alone is sufficient for initiating leukemogenesis. The N-terminus involved in formation of MLL fusion proteins retains the MLL histone methyltransferase activity and therefore it seems that the leukemia-causing molecular mechanism is the aberrant target of epigenetic modifications. Among the genes misregulated by MLL fusion proteins, the most relevant class is the HOXA family, a group of genes fundamental in development, which are normally regulated by MLL and are frequently overexpressed in leukemia [91]. Recently, Nguyen et al [92] have demonstrated that MLL-AF9 fusion protein interact with DOT1L, an H3K79 methyltransferase and this interaction is required for both initiation and maintenance of MLL-AF9–induced leukemogenesis both *in vitro* and *in vivo*. Moreover, through gene expression and chromatin immunoprecipitation analysis the authors demonstrated that mistargeting of DOT1L, result in epigenetic changes at HOXA genes. In fact, according to their results H3K79 methylation and the consequent up-regulation of HOXA genes underlie the molecular mechanism of how DOT1L contributes to MLL-AF9–mediated leukemogenesis. DOT1L also has been associated to the leukemic transformation by MLL-AF10, MLL-ENL and CALM–AF10, presumably through the same mechanism [90,93,94].

Although the involvement of histone demethylases (HDMs) in cancer progression has already been established, there is no much data regarding their role in leukemia. Recently it was found that lysine (K)-specific demethylase 2B (KDM2b), a demethylase specific for H3K36me2, is upregulated in AML and has a critical role both in the initiation and progression of the disease [95].

### AML treatment with epigenetics drugs

There are more than 100 epigenetic agents currently under investigation and a few have received US Food and Drug Administration (FDA) approval in the last decade. According to the web site ClinicalTrials.gov, maintained by the US National Institute of Health and that currently lists 143,954 studies with locations in all 50 states and in 184 countries, there are 1.818 studies for AML. From the total number of studies, 608 are open studies defined by clinical trial web site as: “studies that are currently recruiting participants, will be recruiting participants in the future, or involve drugs that are available for expanded access”. A search for AML and epigenetic retrieved 12 open studies, however searching for DNA methylation and AML or histone and AML retrieved 24 and 48 studies, respectively.

### Clinical drugs targeting DNA methylation

DNA methyltransferase inhibitors have found the earliest and greatest success as prototypical epigenetic agents. In 2004, the DNA methyltransferase inhibitor, azacytidine (VidazaTM), was approved by the FDA for the treatment of myelodysplastic syndrome MDS, on the basis of phase II and III clinical trials. In 2006 a second DNA methyltransferase inhibitor, decitabine (dacogene) received FDA approval also for treatment of MDS. In these studies, treatment with decitabine or azacitidine exhibit a response rate up to 30% that was relatively durable when compared to placebo [96,97]. Azacitidine is now also being evaluated in clinical trials on other malignant diseases such as non-small cell lung cancer and pancreatic cancer [98,99].

Azacitidine and decitabine (5-aza-2 deoxycytidine) are cytidine analogs in which the carbon atom at position 5 in the pyrimidine ring has been replaced by a nitrogen atom. Following cellular uptake, azacitidine and decitabine are converted into their corresponding monophosphates, diphosphates, and triphosphates nucleotides. As nucleotide analogues, they replace cytosine during DNA replication, producing DNA demethylation by inactivation of DNMTs. Despite indiscriminately targeting DNMTs [100], they were found at low dosages to selectively reactivate gene expression with relatively few side effects [101]. Incorporation into the DNA results in the formation of adducts between the DNA and DNMT. Although at high doses, the DNA is not able to recover and cell death occurs, at lower doses the formed adducts are degraded by the proteosome, after which the DNA is restored. DNA synthesis is then resumed in the absence of DNMT, and as a consequence the aberrant DNA methylation pattern can no longer be established in the DNA daughter strands. In this manner, a low dose of azacitidine or decitabine is able to induce re-expression of previously silenced genes [102-104].

### Clinical drugs targeting histone modifications

Several clinical trials of histone deacetylase inhibitors (HDACi) have been conducted in solid and hematological malignancies. Interestingly, until now the results demonstrate a preferential efficacy in hematological malignancies [105]. These compounds are also beginning to be tested in combination therapies, either as chemo sensitizing agents in association with standard chemotherapy drugs or in combination with DNA methyl transferase inhibitors (Table 1). In human, the biological target of HDACi, the histone deacetylases complexes (HDACs), comprise a family of 18 genes sub-divided into 4 classes based on their sequence homology to yeast proteins, sub-cellular localization and enzymatic activities [106]. HDAC belonging to class I (HDAC1, HDAC2, HDAC3 and HDAC8), class IIa (HDAC4, HDAC5, HDAC7 and HDAC9), class IIb (HDAC6 and HDAC10) and class IV (HDAC11) exhibit a Zn dependent enzymatic activity, which is the bases of their interaction with the HDACi currently used. Class III (or Sirtuins) constitute a structurally separate subfamily which exhibit a NAD-dependent enzymatic activity.

**Table 1:** Selected histone deacetylase inhibitors

**Name**	**Chemical class**	**Formula**	**Target**	**Number AML trials**	
				**Open/Total**	**+ Azacytidine open/Total**
Phenylbutyrate	Short fatty acid chain		Pan-inhibitor	0/6	0
Valproic acid	Short fatty acid chain		ClassI/IIa	5/14	1/7
Vorinostat (SAHA)	Hydroxamic acid		Pan-inhibitor	8/25	2/4
Belinostat (PXD101)	Hydroxamic acid		Pan-inhibitor	1/4	0/1
Panobinostat (LBH589)	Hydroxamic acid		Pan-inhibitor	7/11	2/3
Trichostatin A	Hydroxamic acid		Pan-inhibitor	0	0
Givinostat (ITF2375)	Hydroxamic acid		Pan-inhibitor	0	0
Mocetinostat (MGCD0103)	Benzamide		Class I/IV	0/3	0/1
Entinostat (MS275-SNDX275)	Benzamide		Class I	2/7	1/3
Romidepsin (Depsipeptide)	Cyclic tetrapeptide		Class I	0/5	0/1
Trapoxin B	Cyclic tetrapeptide		Class I/IIa	0	0

Chemical structures from http://www.chemspider.com.

HDACi can be classified based on their general chemical structure in short fatty acid, cyclic peptide benzamide and hydroxamic acids (Table 1). These inhibitors differ in potency, pharmacokinetic properties and, more importantly, on their selectivity to inhibition targets. According to their *in vitro* selectivity profile, HDACi are classified in pan HDACi, which inhibit HDAC class I and II (e.g. vorinostat, panobinostat and belinostat), or selective HDACi, which inhibit either HDAC class I (e.g. mocetinostat, entinostat) or class II (e.g. MC1568) [107]. It is generally accepted that particular attention should be paid to HDACi inhibition selectivity in the interpretation of experimental results; although it must be stress that the selectivity profile has been mainly determined *in vitro* and therefore may not reflect the physiological state of HDACs. Moreover, it is still unknown if a selective HDACi could have a better balance among desired and undesired effect than a pan HDACi.

The effects of HDAC inhibitors appear to be to promote G1 or G2/M cell-cycle arrest, as well as apoptosis and cell differentiation. It is important to keep in mind that histone are not the only targets of HDAC, but among their know substrates are also included p53, signal transducer and activator of transcription 3 (STAT3), heat shock protein 90 (hsp 90), and other important proteins and therefore changes in the acetylation status of these other proteins may contribute to the biological effects observed [108].

Only two HDACi have been approved by the FDA: Vorinostat for the treatment of cutaneous T cell lymphoma (CTCL) on October 6, 2006, which has also given encouraging results in a phase II trial for MDS in combination with Idarubicin and Cytarabine, and Romidepsin for CTCL on November 5, 2009. However, there are several more been tested in phase I, II or III for different diseases. Currently, according to Clinical Trials Database (http://www.clinicaltrials.gov), for AML treatment there are ongoing clinical trials for: valproic acid, vorinostat, belinostat, panobinostat and etinostat either alone or in combination with the DNA methylation inhibitor 5-azacytidine (decitabine) (Table 1).

Somehow unexpectedly, HAT inhibitors have also been shown to have some antitumor activity. This contrasts with the global hypoacetylation already seen in many cancers. Naturally occurring drugs such as curcumin, garcinol, and anacardic acid appear to selectively inhibit the acetyl transferases p300, CBP, or PCAF, leading to apoptosis or sensitization to therapies such as radiation [109-111]. Until know no clinical trials have yet been completed with this class of agents.

Epigenetic based therapies have so far focused on the use of DNMTs and HDACs inhibitors, which tend to have more general and widespread effects on gene regulation in the cell. However, if a unique molecular pathway can be identified in diseases caused by epigenetic mechanisms, they will be excellent candidates for the development of more targeted therapies that focus on specific gene targets, individual binding domains, or specific enzymatic activities. Therefore, designing effective targeted therapies depends on a clear understanding of the role of epigenetic mutations during disease progression.

### AML Epigenetics: patent applications and granted patents

In this section we summarize, current information publically available in the international system of Patents of Inventions registered mainly at the World Intellectual Property Office (WIPO, http://www.wipo.int/portal/index.html.en) as well as at the european (http://worldwide.espacenet.com), the japanese (http://www.jpo.go.jp/) and the US (http://patft.uspto.gov/) patents offices. Briefly, patents applications are those still in analysis to define if they will be or not granted, but we include them as a sample of the efforts to develop new technological strategies against AML and its epigenetics causes. Tables 2 and 3 summarize patent documents related to the epigenetic tools currently proposed for AML diagnostic and therapeutic strategies.

**Table 2:** Selected strategies using epigenetic tools for leukemia diagnostic as proposed in the International Database for Patents of Inventions

**Patent document**	**Target gene**	**Method based on**
WO2006060429	HDAC1 gene	Detection of five point mutations in HDAC1: M51L, Q111K, T114A, V157G and a premature stop codon at R34
WO2012078288	DNMT3A	Detection of one or more mutations, mainly at Arg-882
WO2009150229	TET2	Detection of expression levels or mutations in this putative suppressor gene
US2009317801	Genes located on the long arm of chromosome 17 (17q25.3)	Analysis of methylation state of a target gene in this chromosomal region
US2011281270	CEBPA	Detection of bi allelic mutations in CCAAT-enhancer binding protein gene

**Table 3:** Selected compounds proposed as epigenetic tools for leukemia treatment according to the International Database for Patents of Inventions

**Patent document**	**Compound**	**Proposed function**
WO2011143660		BDR4 inhibitor
WO2010009285		HDAC inhibitor
US20080138329		DNMT inhibitor
US2010305059		antiproliferative
US2006106049		HDAC inhibitor
ES2399670		HDAC inhibitor

## Conclusions

Despite recent major advances in our understanding of the genetics of myeloid malignancies, there have been far fewer examples of how these insights have been translated to novel therapies. Epigenetic modifiers provide new targets for therapeutic intervention. Mutations in IDH1 and IDH2 that result in a new enzymatic activity may represent novel, tractable targets for this genetically defined subset of leukemia patients. Likewise, enzymatic activities associated with genes involved in leukemic transformation (including H3K79 methyltransferase activity, histone acetyltransferase activity and other chromatin enzymatic functions) have just recently been explored from a therapeutic standpoint. Worldwide scientific and patents of inventions literature, suggest that advances in our knowledge of the genetics of myeloid malignancies, coupled with an improved understanding of the role of specific epigenetic modifications in leukemogenesis, may probably lead to an increased number and/or progress in the development of therapies that improve outcomes for patients with MPN, MDS and AML in future years. Moreover, there is hope that this knowledge could also give rise to new diagnostic and/or prognostic methods allowing the design of a personalized treatment for each patient, as well as more sensitive procedures to detect the pathologies sooner, which is particularly relevant considering that some of the most common cancer types are largely curable if they are detected early and treated appropriately.

## Competing interests

The authors declare that they have no competing interests.

## Authors’ contributions

FRO and SEG have contributed to data preparation, drafting and revising the manuscript. Both authors read and approved the final manuscript.
